# Prognostic relevance of the Golgi mannosidase MAN1A1 in ovarian cancer: impact of N-glycosylation on tumour cell aggregation

**DOI:** 10.1038/s41416-019-0607-2

**Published:** 2019-10-29

**Authors:** Fabienne Hamester, Karen Legler, Beatrice Wichert, Nicole Kelle, Kathrin Eylmann, Maila Rossberg, Yi Ding, Sascha Kürti, Barbara Schmalfeldt, Karin Milde-Langosch, Leticia Oliveira-Ferrer

**Affiliations:** Department of Gynecology, University Medical Centre Hamburg-Eppendorf, Martinistr. 52, Hamburg, 20246 Germany

**Keywords:** Ovarian cancer, Tumour biomarkers, Glycosylation

## Abstract

**Background:**

Maturation of complex N-glycans involves the action of Golgi mannosidases and plays a major role in cancer progression. We recently showed a favourable prognostic role of α-mannosidase MAN1A1 in breast cancer mainly caused by alteration of certain adhesion molecules.

**Methods:**

We analysed the protein expression of MAN1A1 in ovarian cancer (*n* = 204) using western blot and studied the impact of MAN1A1 itself and of MAN1A1-related glycosylation on the prognostic relevance of two adhesion molecules. Functional consequences of mannosidase inhibition using kifunensine and MAN1A1 knock out were investigated in ovarian cancer cells in vitro.

**Results:**

Patients with high MAN1A1 expression in tumours showed significantly shorter RFS than those with low-MAN1A1 levels. Moreover, high MAN1A1 expression correlated significantly with advanced stage, lymph node involvement and distant metastasis. Further, the glycosylated adhesion molecule ALCAM reveals a significant adverse prognostic effect only in the presence of high MAN1A1 expression. In spheroid-formation assays, mannosidase inhibition and especially MAN1A1 knock out led to strong reduction of tumour cell aggregation.

**Conclusions:**

Our study demonstrates the unfavourable prognostic role of MAN1A1 in ovarian cancer, probably caused by an altered ability of spheroid formation, and the strong influence of this glycosylation enzyme on the prognostic impact of ALCAM.

## Background

Ovarian carcinoma is the gynaecologic tumour with the highest mortality. Since it is asymptomatic in early development, it is mostly diagnosed in advanced stages when tumour dissemination has already taken place. Ovarian cancer (OvCa) therapy includes optimal surgical tumour reduction (debulking) followed by platinum-based combination chemotherapy. Here, the mutation status of genes related with homologous recombination deficiency such as BRCA1/2, has been found recently to predict better response in platinum-treated patients. But up to now these are the only known predictive markers in ovarian cancer and new molecular targets for an individualised therapy are urgently needed.

In contrast to tumours with mainly hematogenic metastasis, i.e. breast cancer, dissemination of ovarian carcinomas occurs by intraperitoneal spread and, partly, through lymphatics, giving rise to retroperitoneal metastatic lesions. In early state OvCa can metastasise also haematogenously to the omentum.^1,2^ Both hematogenic and intraperitoneal metastasis involve changes in cell-cell or cell-matrix interactions accomplished by adhesion proteins. Like most cell-surface proteins these are heavily glycosylated, and the correct glycosylation is essential for their proper function. In cancer cells, aberrant O- and N-glycans are frequently found due to disturbed glycosylation and aberrant expression of glycosylation enzymes.^3^

N-glycosylation is a complex process, which leads to addition of glycan structures to the amino group of asparagine residues during translation. It starts with synthesis of a dolichol-bound oligosaccharide precursor in the endoplasmic reticulum (ER), consisting of 14 sugar moieties, among them nine mannose residues. This oligosaccharide is then transferred to a suitable asparagine residue (Asn-X-Ser/Thr) within the nascent polypeptide by the oligosaccharyltransferase (OST) protein complex. Now, the correct folding and secretion of the glycoprotein depends on trimming of the glycan precursor in the ER and Golgi. After removal of three glucose residues by glucosidases and one mannose by ER mannosidase MAN1B1, the glycoprotein is transferred to the Golgi with its N-glycans containing eight mannose residues, termed high-mannose glycans. Generally, additional mannose residues are then cleaved by Golgi mannosidases which is the prerequisite of formation of complex or hybrid glycans.^4^ The mannosidase MAN1A1, together with MAN1A2 and MAN1C1, belongs to the GH47 (Glycosidase Hydrolase) Golgi mannosidase I (Golgi MI) subfamily,^5^ which cleaves alpha-1,2 bound mannose sugars from high-mannose glycans (Man8–9GlcNAc2) resulting in 5-mannose glycans (Man5GlcNAc2).^6^ Inhibition of Golgi mannosidases has been previously described to result in increased high-mannose glycans which modulates cellular functions, including cell adhesion^7,8^ (Supplementary Fig. S1).

Ovarian and breast cancer are the most important female malignancies, but they strongly differ in their metastatic behaviour including the role of cell adhesion. In prior studies on breast cancer tumour samples, high MAN1A1 mRNA or protein expression was associated with prolonged recurrence-free (RFS) and overall survival (OAS) of the patients as well as significantly less lymph node involvement or brain metastasis, pointing to a tumour-suppressor function of this enzyme.^9,10^ Low-MAN1A1 expression in tumour cells resulted in significantly increased adhesion to endothelial cells in vitro suggesting a role of N-glycans in hematogenic metastasis.^9^ Based on these results on breast cancer, we were interested in the role of the mannosidase MAN1A1 in OvCa, which exhibits a fundamentally different mode of tumour progression, mainly due to intraperitoneal spread.

## Methods

### Patient cohort

MAN1A1 protein expression was analysed in 204 ovarian tumour tissue samples obtained during surgery in the University Medical Centre Hamburg-Eppendorf (UKE). Of these, 176 cases were from primary ovarian carcinomas, 12 samples from recurrent ovarian cancer, 12 from tumours of low malignant potential (LMP, Borderline tumours) and four from benign cystadenomas. The clinical and histological characteristics of the primary carcinomas are shown in Table 1. Patients included in this retrospective study were treated between 1998 and 2012. Informed consent for the scientific use of tissue materials, which was approved by the local ethics committee (Ethik-Kommission der Ärztekammer Hamburg, #OB/V/03), was obtained from all patients. The study was performed in accordance to the principles of the declaration of Helsinki and REMARK criteria.^11^ No radiotherapy, neoadjuvant chemotherapy or endocrine therapy had been administered before surgery.

**Table 1:** Cohort description (*n* = 176)

		patient number (%)
Age at diagnosis (y)	Mean (median)	59.2y (61 y)
Histological Type	Serous-papillary	148 (84.0)
	Endometrioid	10 (5.7)
	Mucinous	4 (2.3)
	Others/unknown	14 (8.0)
FIGO stage	FIGO IA-IC	8 (4.6)
	FIGO IIA-IIB	6 (3.4)
	FIGO IIIA-IIIC	125 (71.0)
	FIGO IV	37 (21.0)
Nodal involvement	Negative	44 (25.0)
	Positive	101 (57.4)
	Unknown	31 (17.6)
Grading	G1	9 (5.1)
	G2	46 (26.1)
	G3	117 (66.5)
	Unknown	4 (2.3)
Distant metastasis	Negative	138 (78.4)
	Positive	37 (21.0)
	Unknown	1 (0.6)
Tumour residuum after surgery	Not macroscopically visible	122 (69.3)
	<1 cm^3^	33 (18.8)
	>1 cm^3^	19 (10.8)
	Unknown	2 (1.1)
Adj. Chemotherapy	Carboplatin/Paclitaxel (Taxol)	127 (72.2)
	Other regimens based on carboplatin	35 (19.9)
	Others	14 (7.9)
Recurrence	Yes	114 (64.8)
	No	57 (32.4)
	Unknown	5 (2.8)
Survival at last follow-up	Alive	83 (47.2)
	Dead	93 (52.8)
Recurrence-free survival (months)	Mean (median)	27.4 (16)
Overall survival (months)	Mean (median)	38.4 (30.5)

### Ovarian cancer cell lines

Cultivation and protein extraction from the human OvCa cell lines SKOV3 (obtained from American Type Culture Collection, Manassas, VA, USA) and OVCAR8 (a kind gift of Dr Volker Assmann, Institute of Tumour Biology, UKE, Hamburg, Germany) were performed as described.^12,13^ The cell line OAW42 (a kind gift of the Institute of Anatomy, UKE, Hamburg, Germany) was cultured in Modified Eagle’s Medium (Gibco, Grand Island, NY) supplemented with 10% fetal bovine serum. Cell lines were recently authenticated and are periodically tested for mycoplasma contamination.

### CRISPR/Cas9-MAN1A1 knock out

CRISPR/Cas9-MAN1A1 knock out (k.o.) in OVCAR8 cells was performed according to the plasmid-based procedure described by Ran et al.^14^ Guide sequences were selected with the provided online CRISPR-Design-Tool. Primers used for sgRNA oligo insert construction can be found in supplementary material and methods file. sgRNA oligos were cloned into pSpCas9(BB)-2A-GFP plasmid (Addgene plasmid ID: 48138). OVCAR8 cells were transfected with MAN1A1-specific CRISPR/Cas9 plasmid as well as empty vector (e.v.) by using Lipofectamine2000 reagent (Thermo Fisher Scientific Inc., Waltham, Massachusetts). Single-Cell-Colonies were assessed using FACS Sorting method und MAN1A1 k.o. was checked via western blot analysis and sequencing of genomic DNA (Supplementary Fig. S2).

### Western blot analysis

Protein extraction from tumour samples and polyacrylamide gel electrophoresis were performed as described before.^15^ Briefly, equal amounts of protein (20 μg) of each sample were loaded per well and equal loading was verified by immunoblotting with GAPDH antibody. As positive control, a protein extract from SKOV3 was loaded in each gel. MAN1A1 was detected by incubation with the anti-α-1,2-mannosidase-IA antibody. Blots were visualised by chemiluminescence reagent (Supersignal West Pico Chemiluminescent Substrate, Thermo Scientific) using Fuji Medical X-Ray Film (Fuji-film Corporation). Band intensities were quantified by densitometry (GS-700 Imaging Densitometer, Bio-Rad, Hercules, California) and calculated as percent-intensity of SKOV3 (set as 100 %) after correction for equal GAPDH loading. The optical densities of both bands (72/60 kDa) were quantified separately as well as together and calculated in relation to the 72 kDa band of SKOV3.

In addition to MAN1A1, expression of the Activated leukocyte cell adhesion molecule (ALCAM/CD166), the Intercellular adhesion molecule 1 (ICAM1) and Integrin β4 were analysed. β- Actin as well as GAPDH expression was used as loading control.

For lectin blots, kifunensine-treated (10 µM) and non-treated whole protein lysates (10 µg) and membrane fractions from SKOV3, OVCAR8 and OAW42 cells were prepared. The membranes were blocked 2 h in carbo-free-blocking solution (Vector Laboratories, Burlingame, CA, USA) and then incubated with biotinylated ConA (con-canavalin A) or PHA-E (*Phaseolus vulgaris erythroagglutinin*). Lectins were detected with HRP-conjugated streptavidin (ABC reagent, Vector Laboratories) and developed on X-ray film using chemiluminescence reagent. α-Tubulin served as loading control. Additional antibody information is listed in the supplementary material and methods file.

### Treatment with kifunensine

OVCAR8 cells were incubated with mannosidase inhibitor kifunensine (Sigma–Aldrich Chemie GmbH) in three different concentrations (1, 10 and 50 µM) for 72 h in serum-reduced medium (5% (v/v) FCS), and functional assays were subsequently performed.

### Subcellular fractionation of cells

Protein extraction of kifunensine-treated cells and untreated controls was performed using Qproteome Cell Compartment Kit (Qiagen, Hilden, Germany) according to the manufacturer´s instructions. The protein levels of ALCAM in resulting subcellular fractions were investigated by using western blot analysis as described before with 10 µg protein per lane. α-Tubulin was used as a loading control for whole lysate and cytoplasm, whereas Lamin A/C was used for the nuclear fraction. For quantification, the protein yields in the four fractions were measured and total ALCAM amounts within these fractions were calculated (*n* = 3).

### Spheroid-formation assay

Cell spheroids were generated on 2% agarose-coated (Seakem^®^ GTG^®^, Lonza Group AG, Basel, Switzerland, dissolved in Dulbecco´s Phosphate Buffered Saline) 96-well plates by seeding 3 × 10^3^ cells in 100 µl full medium per well. The spheroid-formation ability of the cells was determined by comparing spheroid size after three different time points using light microscopy and camera (Axiovert 40 C, Carl Zeiss AG, Leica DFC320, Wetzlar, Germany). Each stimulation with kifunensine was performed in triplicate (*n* = 3). Images are representative of three independent experiments.

### Migration assay

Cells were seeded with 1.5 × 10^5^ per well in six-well plates and cultured until confluence. A wound healing assay was performed as described previously.^13^ The migration potential of the cells was determined by analysing the wound area at different time points (0, 48, and 72 h) with ImageJ Wound Healing Tool (Wayne Rasband, National Institute of Health). Each stimulation was performed in triplicate (*n* = 3). Images are representative of three independent experiments.

### Proliferation assay

Cell proliferation was analysed using the Cell Proliferation Kit I (MTT) (Roche Applied Science, Mannheim, Germany) in medium containing 1% (v/v) or 10% (v/v) FCS. A total of 5 × 10^3^ kifunensine-treated cells (1, 10 and 50 µM for 72 h) were seeded per well into a 96-well-plate in 100 µl medium. Cell viability was quantified after 0, 24 and 48 h by measuring the absorbance of the supernatant at 540 nm and 24 h and 48 h values were related to those at 0 h. Each stimulation was performed in quadruplicates (*n* = 4). Images are representative of three independent experiments.

### Cytotoxicity assay

OVCAR8 cells were seeded (5 × 10^3^ cells per well) in 96-well plates and treated with cisplatin (Neocorp AG, Weilheim, Germany) in three different concentrations (1, 10 and 50 µg/ml) for 24 h. Cell viability was determined using Cell Proliferation Kit I (MTT) (Roche Applied Science, Mannheim, Germany) as described above. Each stimulation was performed in quadruplicates (*n* = 4). Images are representative of three independent experiments.

### Statistics

Statistical analysis was conducted using SPSS software Version 24 (IBM SPSS Statistics, Armonk, NY, USA). Correlations between the MAN1A1 mRNA and protein expression values were examined using two-sided Pearson tests. For further statistical analysis all tumour cases were first divided into four groups of similar size (quartiles Q1–Q4) representing low, moderate/low, moderate/strong and strong MAN1A1 expression, then further combined to two groups with the median or the 75 % point as cut-off.

Chi-square-tests were used to examine the correlations between MAN1A1 expression and tumour type or clinicopathological factors in primary carcinomas. For prognostic parameters, the following groups were compared: histological grading (G1/2 vs. G3), FIGO stage (I/II vs. III vs. IV), nodal status (positive vs. negative); histological subtype (serous vs. others), residual disease after debulking surgery (none vs. <1 cm vs. >1 cm). Survival curves were plotted by Kaplan-Meier analysis. Differences between survival curves were evaluated by Log-Rank-Tests. Hazard ratios were calculated by uni- or multivariate Cox regression analysis.

For all in vitro assays, cells in each group were plated in triplicates or quadruplicates, and each experiment was performed three times (*n* = 3). Statistical significance was determined using unpaired two-tailed Student’s *t*-tests. The assumption of homogeneity of variance was tested using Levene's Test of Equality of Variances (*p* > 0.05). Results are given as mean ± s.d. or s.e. Probability values less than 0.05 were regarded as statistically significant.

## Results

### MAN1A1 expression in different ovarian tumour types

**Fig. 1:**
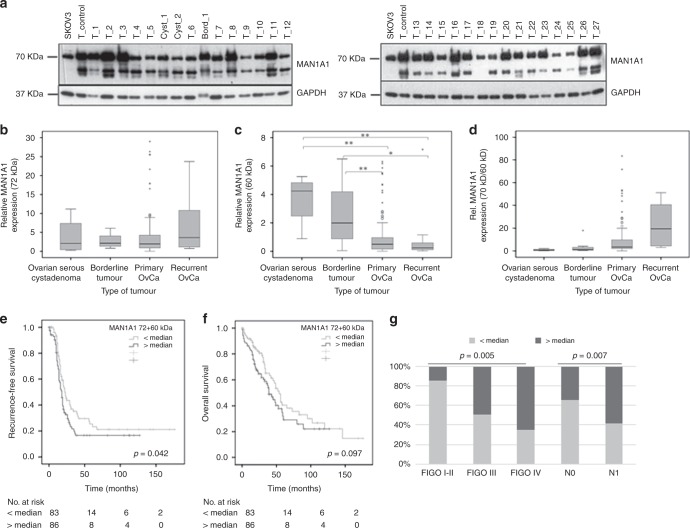
MAN1A1 protein expression in ovarian tumours and its impact on patient survival. **a** Representative western blot results of MAN1A1 expression in ovarian carcinomas (T_), borderline tumours (Bord_) and benign cystadenomas (Cyst_). Protein extract from the ovarian cancer cell line SKOV3 as well as protein extract from a selected tumour tissue sample (T-control) were included in each gel as internal controls. As loading control, GAPDH expression is shown. **b**, **c** Box plots showing MAN1A1 expression relative to the control cell line in different ovarian tumour types (**b** 72 kDa-band; **c** ca. 60 kDa-bands). **d** Box plots showing the ratio of the two measured MAN1A1 forms (70 kDa/ca. 60 kDa) in different tumour types. Significant differences after t-tests are marked by asterisks (*p < 0.05; **p < 0.01). Whiskers represent the minimum and maximum rating values, circles represent outliers and asterisks represent extreme outliers of each box plots. **e**, **f** Kaplan-Meier analysis and Log-rank test showing a significant correlation of high MAN1A1 (72 kDa + 60 kDa) protein expression (>median) with shorter recurrence-free survival and a trend towards overall survival. **g** Correlation analysis by Chi-square-test showing a significant association of high MAN1A1 (72 kDa + 60 kDa) protein expression (>median) with higher FIGO stages and nodal involvement

A representative western blot of MAN1A1 and GAPDH is shown in Fig. 1a. MAN1A1 expression in SKOV3 control cells was defined as 100% for statistical analysis. MAN1A1 was strongly expressed and consistently detected around 72 kDa as expected in all tumours (mean relative expression 376%; range 1.1–2898%). An additional band appeared around 60 kDa in most tumour samples (mean relative expression 103%; range 0.4–717%), partly in form of a double band, whereas this could not be detected in SKOV3. No additional bands were detected neither for the control cell line nor for the tissue samples. The correlation between mRNA level and protein level was analysed in a small set of OvCa samples (*n* = 37). Here, mRNA levels correlated significantly with corresponding protein levels including 72 kDa bands or 60 kDa bands or 72 kDa + 60 kDa bands (72 kDa: Pearson correlation (r) 0.586; *p* = 0.000; 72 kDa + 60 kDa: r = 0.493; *p* = 0.002; 60 kDa: r = 0.379; *p* = 0.021). Both bands correlated strongly with each other by Pearson tests (r = 0.672, *p* < 0.001) in the OvCa cohort. All densitometric analysis was conducted with expression levels of the expected band at 72 kDa, the cumulated expression values of all bands, and the 60 kDa bands alone.

MAN1A1 protein expression was detected and compared in benign cystadenomas, borderline tumours and primary or recurrent ovarian carcinomas. Mean relative expression of the major band (72 kDa) in these four groups were 389, 273, 364 and 651%, respectively (median values: 209, 213, 195 and 362%; interquartile-ranges: 8.9, 2.9, 3.3 and 10.7; Fig. 1b), with no statistically significant differences in t-tests. Regarding expression of the 60 kDa band in these four tumour types, we observed mean values of 366, 252, 88 and 90% (median values 424, 199, 50 and 24%; interquartile-ranges: 3.3, 3.7, 0.8 and 0.5; Fig. 1c). Thus, there was a significantly higher expression of the 60 kDa band in benign cystadenomas or borderline tumours compared to primary or recurrent invasive carcinomas, whereas the 72 kDa protein predominates in carcinomas (Fig. 1b) and in SKOV3 only the larger variant is detectable (Fig. 1a). This trend is clearly visible in Fig. 1d, where the ratio of both bands is shown for the different ovarian tumour types. Here, we found significantly increasing values with higher malignancy (mean values 0.9, 3.0, 21.1 and 22.3; median 0.8, 1.2, 3.5 and 19.5%; interquartile-ranges: 1.6, 2.8, 7.8 and 37.0; Fig. 1d). No significant differences were detected regarding the intensity of the combined MAN1A1 bands (Supplementary Fig. S3).

### Correlations with clinical and histological parameters and prognostic relevance

For correlation analysis between MAN1A1 expression and clinical/histological parameters all primary carcinomas were divided into two groups of equal size with MAN1A1 (72 kDa) expression below or above median. Interestingly, using Chi-square tests high MAN1A1 protein levels correlated significantly with advanced stage and the presence of distant metastasis, whereas there was no association with histological grading and lymph node involvement (Supplementary Table S1). In addition, optimal debulking results with no macroscopically visible residual tumour were achieved significantly less frequently in cases with high MAN1A1 expression. In the same way, associations of the second band (60 kDa) with prognostic parameters were analysed. Here, we only found a significant correlation of high expression with distant metastasis (Supplementary Table S1). Regarding the combined bands, significant associations with advanced stage, positive lymph node status and distant metastasis were found (Supplementary Table S1, Fig. 1g).

By Kaplan-Meier and univariate Cox regression analysis, high expression of the combined MAN1A1 bands (72 + 60 kDa) was shown to correlate with a significantly shorter RFS (Fig. 1e, Cox regression: *p* = 0.047; Hazard ratio 1.46; 95% CI 1.01–2.11), but not with OAS (Fig. 1f). Kaplan-Meier analysis with quartiles showed the same trend but with weaker significance, probably due to the lower case-number per group (Supplementary Fig. S4A and S4D). Similar results were also obtained for the 72 kDa MAN1A1 band, with a significant association of high expression with shorter RFS (*p* = 0.034; Hazard ratio 1.48; 95% CI 1.02–2.41, Supplementary Fig. S4B and S4E), but not OAS (Supplementary Fig. S4C and S4F). Yet, here the correlation with OAS reached statistical significance if another cut-off was used (lower 75% vs. upper 25%; *p* = 0.022; HR 1.68; 95% CI 1.07–2.63). High levels of the 60 kDa band were only associated with a significantly shorter OAS (Supplementary Fig. S4F, Cox regression: HR 1.57, 95% CI 1.04–2.37; *p* = 0.030). In multivariate analysis including the most important prognostic parameters, FIGO stage and residual tumour after surgery, MAN1A1 expression (72 kDa, 60 kDa or combined) lost its prognostic significance.

### Influence of the mannosidase inhibitor kifunensine on adhesion proteins in ovarian cancer cells

**Fig. 2:**
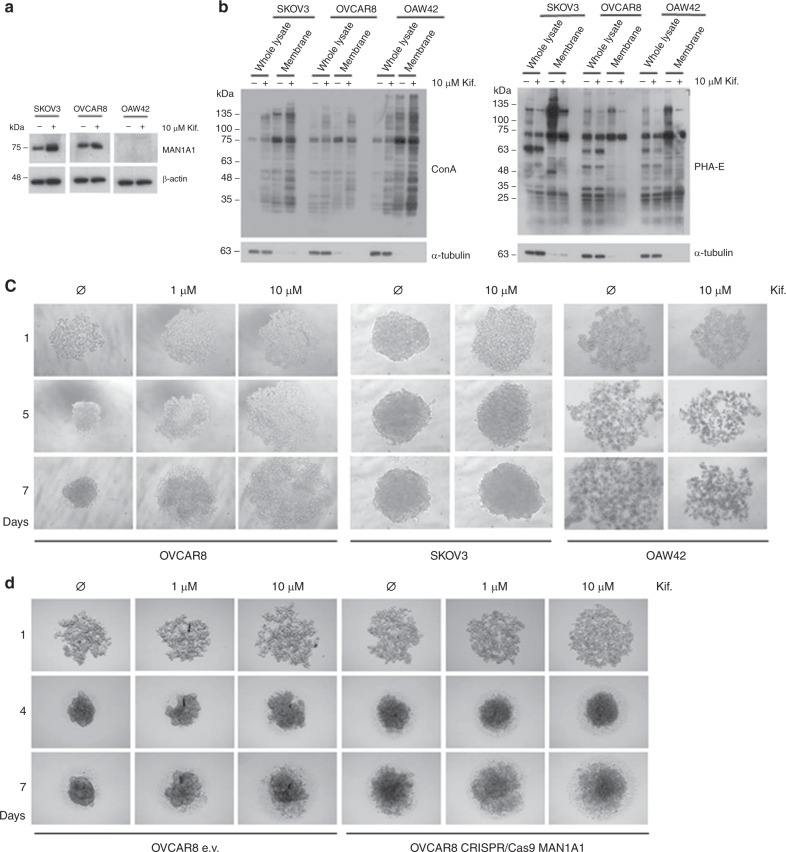
Influence of mannosidase inhibition on the spheroid-formation ability of ovarian cancer cells. **a** Western blots showing the endogenous MAN1A1 expression in the ovarian cancer cell lines SKOV3 and OVCAR8 and a lack of MAN1A1 in OAW42 cells. The treatment with 10 µM kifunensine only effects MAN1A1 expression in SKOV3 cells. As a loading control, β-Actin is shown. **b** Western blots showing different reactivity of whole protein lysates and protein lysates from membrane fractions of SKOV3, OVCAR8 and OAW42 cells to ConA and PHA-E lectins with and without kifunensine treatment. For the whole lysate α-Tubulin was used as a loading control. **c** The spheroid-formation assay shows a strong effect of kifunensine treatment (1 and 10 µM) on the tumour cell aggregation ability of the cell line OVCAR8 on days 1, 5 and 7. A similar but weaker effect is visible with the cell line SKOV3, especially on day 1. OAW42 cells failed to form compact tumour spheres and kifunensine treatment had no remarkable effect on its spheroid-formation ability (*n* = 3). **d** MAN1A1 k.o. (CRISPR/Cas9) in OVCAR8 cell line results in an altered spheroid-formation ability compared to OVCAR8 empty vector (e.v.) after 4 and 7 days. The treatment with kifunensine (1 and 10 µM) has a similar but weaker effect on aggregation of spheroids in MAN1A1 k.o. cells (*n* = 4)

Since we found a clear correlation of high MAN1A1 protein levels—presumably resulting in high amounts of complex N-glycans—with high tumour aggressiveness, we assumed that expression of this mannosidase might influence the biological properties of OvCa cells thereby affecting tumour progression and metastasis. First, MAN1A1 protein expression was analysed in SKOV3, OVCAR8 and OAW42. Here, a high endogenous MAN1A1 expression was found in the first two cell lines, whereas it was not detectable in OAW42 (Fig. 2a). In addition, treatment with 10 µM kifunensine results in a slightly increase of MAN1A1 expression only in SKOV3 cells.

Kifunensine was used to analyse the importance of N-glycosylation in OvCa progression in vitro. The inhibitor was first tested for its impact on the biosynthesis of N-glycans using the lectins ConA and PHA-E, which bind preferentially high-mannose-type and complex-type N-glycans, respectively. As expected, we confirmed higher levels of total high-mannose glycans in SKOV3, OVCAR8 and OAW42 cells after treatment with 10 µM kifunensine, especially in the membrane fraction. Accordingly, a strong decrease in complex-type N-glycans were detected in treated cells (Fig. 2b).

In contrast to the majority of tumour entities that metastasise haematogenously, OvCa spreads mainly by peritoneal and/or lymphatic dissemination. This pathway is associated with malignant ascites which predominantly consists of multicellular spheroids. In spheroid-formation assays, non-pre-treated OVCAR8 and SKOV3 cells gave rise to compact, rigid and globular spheroids that were resistant to gentle agitation or physical transfer after 48 h. In contrast, OAW42 cells, which do not express MAN1A1, did not form compact aggregates (Fig. 2c). Interestingly, treatment with kifunensine strongly inhibits OVCAR8 spheroid formation already at low concentration (1 µM), and this effect remains clearly visible after 7 days incubation time. We observed a similar but weaker effect for SKOV3 cells, especially in the first phase of the spheroid formation, yet this effect vanished after day 5. For OAW42 kifunensine treatment did not show any remarkable effect on the spheroid-formation ability (Fig. 2c).

To clarify the role of MAN1A1 in cell aggregation ability we performed spheroid-formation assays with MAN1A1 k.o. in OVCAR8 cells. Here, MAN1A1 deficiency leads to a weaker spheroid-formation ability compared to control cells (e.v.) and the kifunensine treatment has barely no effect on MAN1A1 knob cells (Fig. 2d).

### Influence of mannosidase inhibition on proliferation, chemosensitivity and migration of ovarian cancer cells

In order to analyse the impact of mannosidase activity on further cellular properties of ovarian cancer, OVCAR8 cells were treated with kifunensine and subsequently tumour cell proliferation, chemosensitivity as well as cell migration were evaluated.

**Fig. 3:**
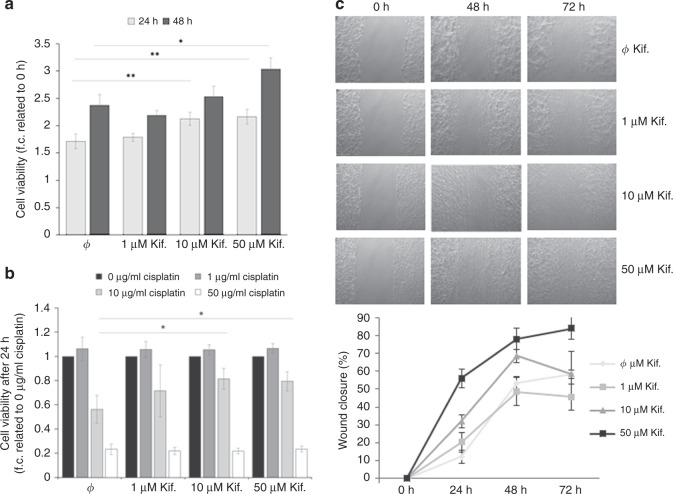
Influence of mannosidase inhibition on proliferation, platin-sensitivity and migration of ovarian cancer cells. **a** Proliferation of OVCAR8 cells in normal growth medium after treatment with 1, 10 and 50 µM kifunensine. Cell viability as determined by MTT test after 24 and 48 h and plotted as % values to 0 h. **b** Viability of OVCAR8 cells treated with 1, 10 and 50 µM cisplatin after pre-treatment with kifunensine. Cell viability was determined by MTT test after 24 h and plotted as normalised values against cisplatin untreated cells (value = 1). **c** Impact of kifunensine treatment on OVCAR8 cell migration ability as determined by a wound healing assay. Using ImageJ Wound Healing Tool, gap closure was quantified and represented as the percentage of cleared area remaining at 24, 48 and 72 h after the initial scratch. Representative results from one of three independent experiments (*n* = 3) and quantification are shown. Values are means ± s.d. *p < 0.05, **p < 0.005

Treatment with 10 µM and 50 µM kifunensine led to a slight increase of OVCAR8 cell growth after 24 h, whereas after 48 h the effect was only noticeable at the highest concentration (Fig. 3a). Further, cell viability analysis after exposure to the cytostatic drug cisplatin for 24 h showed decreased chemosensitivity in cells pre-treated with indicated kifunensine concentrations in comparison with untreated cells. This effect could only be observed after treatment with 10 µg/ml cisplatin, whereas no significant differences were noted with lower (1 µg/ml) or higher (50 µg/ml) cisplatin concentrations (Fig. 3b).

In contrast to the weak effect of kifunensine treatment on OvCa cell proliferation and platin-sensitivity, a clear increase of tumour cell migration after treatment with 10 and 50 µM kifunensine was found in wound healing assays (Fig. 3c).

### Influence of MAN1A1 expression on the glycosylation pattern and prognostic value of cell adhesion molecules

**Fig. 4:**
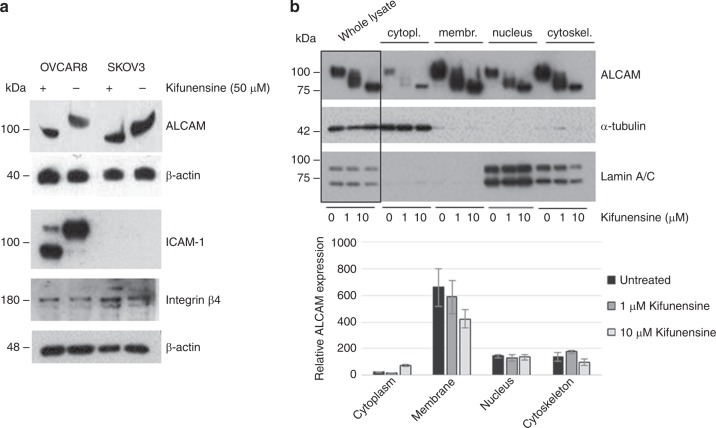
Effect of mannosidase inhibition on the molecular mass and subcellular localisation of cell adhesion molecules. **a** Western blot analysis showing a mass shift in ALCAM, ICAM-1 and to a less extent in Integrin β4 after treatment of OVCAR8 and SKOV3 cells with 50 µM kifunensine. **b** Representative Western blots (upper side) from one of three independent experiments (*n* = 3) showing the effect of kifunensine treatment (1 and 10 µM) on the molecular weight of ALCAM in the whole lysate as well as in each of the fractions (cytoplasm, membrane, nucleus and cytoskeleton). α-Tubulin was used as a loading control for whole lysate and cytoplasmic fraction, whereas Lamin A/C was used for the nuclear fraction. Quantification of the western blot analysis (lower side, *n* = 3) shows a decrease of ALCAM protein amount in the cell membrane fraction and a slightly increase in the cytosolic fraction after kifunensine treatment. Values are means ± s.e

Our observation that mannosidase inhibition affects tumour cell aggregation led us to the assumption that the blockade of the N-glycan maturation via kifunensine treatment might result in the production of aberrantly glycosylated cell adhesion molecules (CAMs), with altered functional properties. The impact of the mannosidase inhibitor on the glycosylation pattern of certain CAMs could be corroborated by western blot analysis of kifunensine-treated versus untreated cells. Here, the N-glycosylated adhesion molecules ALCAM and ICAM-1 showed a molecular mass shift in OVCAR8 and SKOV3 cells after treatment, whereas for Integrin β4 no effect on the molecular weight in OVCAR8 cells and a very slight shift in SKOV3 cells could be noted (Fig. 4a). Further, cell fractionation experiments corroborated the impact of kifunensine on the glycosylation pattern of membranous ALCAM in OVCAR8 cells, which is ultimately responsible for its adhesive function (Fig. 4b). In addition, we could observe a dose-dependent decrease of ALCAM in the membrane fraction and a simultaneous increase in the cytoplasm after treatment with 1 and 10 µM kifunensine (Fig. 4b).

**Fig. 5:**
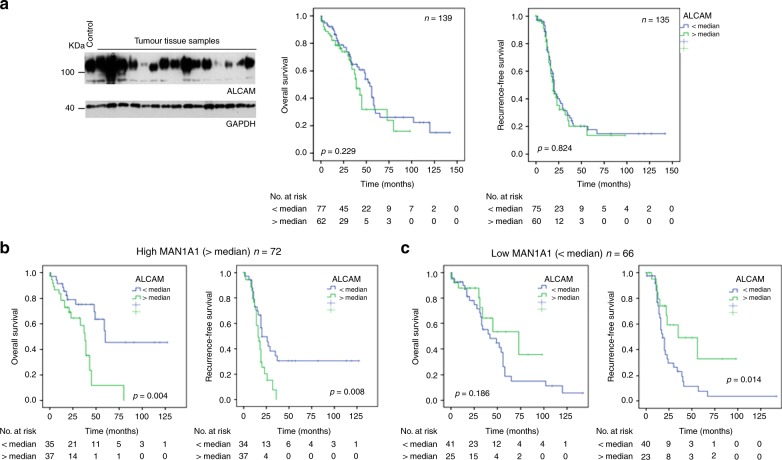
Influence of MAN1A1 expression on the prognostic role of ALCAM in ovarian cancer tissue samples, based on western blot data for both proteins. **a** Exemplary Western blot showing ALCAM expression in ovarian cancer tissue samples and GAPDH as a loading control. Kaplan-Meier analysis and Log-rank test shows no significant correlation of ALCAM with recurrence-free and overall survival. **b**, **c** Regarding the ALCAM expression data, the cases were divided into two groups (</> median expression) for Kaplan-Meier analysis and Log-rank tests, stratified for low (< median) or high (> median) MAN1A1 mRNA expression (all bands). High ALCAM expression correlated significantly with shorter RFS only in cases with a higher mannosidase MAN1A1 expression (**b**). In contrast, high ALCAM levels were associated with longer RFS in tumours with low-MAN1A1 expression (**c**)

Next, we aimed to clarify whether the expression level of MAN1A1 in tumours could impact the prognostic value of ALCAM and ICAM-1. Therefore, western blot analysis with both molecules were performed in carcinomas of the previously mentioned cohort followed by survival analysis with the obtained expression data. We did not find any prognostic significance regarding RFS or OAS for ALCAM (Fig. 5a) and ICAM-1 (Supplementary Fig. S5A, B). Then, separate survival analysis was performed in tumours with high and low-MAN1A1 expression. This stratification led to interesting results regarding the prognostic impact of ALCAM expression: In tumours with high MAN1A1 expression (probably leading to high amounts of complex N-glycans), high ALCAM protein expression was significantly associated with a shorter OAS and RFS (Fig. 5b). Surprisingly, the opposite result was found in tumours with low-MAN1A1 protein levels (probably leading to higher amounts of high-mannose N-glycans), where high ALCAM levels were rather associated with a better prognosis (Fig. 5c). This difference was observed for total MAN1A1 expression (all bands), and similarly for the 70 kDa and 60 kDa MAN1A1 bands (Supplementary Fig. S6). In multivariate Cox regression analysis including clinical stage and residual tumour after surgery, ALCAM lost its prognostic significance in groups with high or low-MAN1A1 expression. In the same way, we studied the potential influence of MAN1A1 expression on the prognostic influence of ICAM1. Here, stratification according to MAN1A1 protein levels did not lead to any significant correlation with RFS or OAS (Supplementary Fig. S5C, D).

## Discussion

Golgi mannosidases are necessary for the maturation of complex N-glycans, and among this group of enzymes, MAN1A1 seems to play a major role in cancer cells. In a search for relevant glycosylation enzymes in breast cancer, MAN1A1 was one of eight genes whose mRNA expression was significantly associated with prognosis in two different cohorts.^10^ Experimental studies suggest that this prognostic role is at least partly brought about by alteration of the adhesive properties of breast cancer cells.^9^ Moreover, in hepatocellular carcinoma (HCC) cell lines, MAN1A1 was among those genes with differential expression between metastatic versus non-metastatic cells.^16^ In our present study we show that this mannosidase is also a significant prognostic indicator in OvCa. Yet, in contrast to the other entities, where high expression is associated with a less malignant phenotype, high MAN1A1 expression in OvCa is associated with a shorter RFS, suggesting an oncogenic effect of this gene.

Similar to breast cancer, western blot analysis of ovarian tumour tissue generally detects not only the expected 72 kDa band of MAN1A1, but also one or two bands of around 60 kDa. Interestingly, this smaller MAN1A1 variant is mainly found in non-invasive tumours like cystadenomas and LMP tumours, whereas in carcinomas, the 72 kDa protein predominates and in the OvCa cell lines, only the larger variant is detectable. To date, we cannot explain the presence of this second MAN1A1 band, which might be an as yet unknown splice variant or result from proteolytic processing or impaired glycosylation. Yet, regarding the strong correlation between expression values of both bands and between protein and mRNA expression values, we have no doubt that both are MAN1A1 protein variants.

In ovarian cancer cells as well as ascites or extracellular vesicles from OvCa cells, high amounts of O- and N-glycans have been found by lectin studies or glycan mass spectrometry.^17,18^ Interestingly, in ascites fluid a high enrichment of proteoglycans such as lumican, agrin, versican and dystroglycans were observed carrying a significant amount of O-linked glycosylation.^18^ Further, extracellular vesicles (EVs) of OvCa cells were enriched in glycoprotein galectin-3 binding protein (LGALS3BP) and comprise sialylated complex N-glycans.^17^ Along with our findings the inhibition of N-glycosylation via kifunensine changed composition of EVs and induced a decrease of several glycoproteins like CD63 and L1CAM in EVs.^17^

Similar to other tumour entities, OvCa cells frequently show an aberrant glycosylation pattern of membrane proteins, which might affect their functional properties. In a study on OvCa cells and normal ovarian epithelial cells, Anugraham found increased levels of “bisecting N-glycans”, sialylated N-glycans and “N, N-diacetyllactosamine” type N-glycans in cancer cells.^19^ Forced expression of the sialyltransferase ST6GAL1, which catalyses sialylation of N-glycans resulted in increased cell viability and platin-resistance in OvCa cells.^20^ Similarly, inhibition of N-linked glycosylation by resveratrol triggers ER-stress-mediated apoptosis in OvCa cells.^21^

Our in vitro analysis showed a weak effect of reduced mannosidase activity on OvCa cell proliferation and chemosensitivity. In addition, we found a clear increase in cell migration after mannosidase inhibition, suggesting that accumulation of high-mannose and simultaneous decrease in complex N-glycans might promote cell motility. Yet, this finding does not correspond to the favourable characteristics of low-MAN1A1 in tumours and suggests a minor impact of this feature on OvCa progression. Indeed, peritoneal cancer cell dissemination does not depend on cell motility, but mainly occurs by passive transport within the intraperitoneal fluid.

In contrast, the fact that inhibition of α-mannosidases and especially loss of MAN1A1 impaired the formation of tumour cell aggregates is in line with the clinical data. Multicellular tumour spheroids, which mimic the cellular situation in the malignant ascites and represent the main source of intraperitoneal spread, exhibit enhanced survival and drug resistance compared to single cells as demonstrated with primary ascites-derived epithelial OvCa cells^22^ as well as with OvCa cell lines.^23,24^ Recently, it has been shown that multicellular spheroids found in the ascites arise preferentially from collective detachment. These tumour cell aggregates have a potent survival advantage over single cells and are capable of seeding intra-abdominal metastases thereby retaining the heterogeneity from the primary tumour.^25^

Our results show a MAN1A1-dependent spheroid-formation ability in ovarian cancer cells. Cell lines with high endogenous MAN1A1 expression form cell aggregates in vitro, whereas those with a lack of MAN1A1 -like OAW42- have no ability to build compact spheroids. Further, MAN1A1 inhibition, using the α-mannosidase inhibitor kifunensine, or MAN1A1 k.o. leads to an impaired tumour cell aggregation. We observed a similar kifunensine effect in two different cell lines but in a different extent, even though they express MAN1A1 at similar levels. Here, this effect seems to be not only dependent on the MAN1A1 expression level but also on the expression and activity of other α-mannosidases. This aspect has to be taken into consideration in further studies including patient material.

Based on our present results we hypothesise that OvCa cells require an intact N-glycosylation machinery, including high MAN1A1 activity, in order to preserve the functionality or the subcellular localisation of certain CAMs involved in tumour cell-cell adhesion and consequently to sustain tumour cell aggregation. Several studies have demonstrated the influence of N-glycosylation on protein function. In example, ICAM-1 and JAM-A glycosylation strongly affects cell adhesion and cell migration of endothelial cells and influences protein half-life.^7,26^ In this context, impaired N-glycosylation affects protein transport and cellular localisation of certain glycoproteins. Particularly, glycoproteins containing high-mannose structures, which are regarded as the less mature glycan forms, might be retained in the ER and are more quickly degraded by the proteasome, thereby decreasing their cell-surface levels.^27,28^ These findings are in line with our results on the cellular localisation of ALCAM after treatment with the mannosidase inhibitor and might be also valid for further CAMs in OvCa cells.

We are aware that the effects observed in vitro using kifunensine cannot be exclusively attributed to the inactivation of MAN1A1 as this substance inhibits further mannosidase I enzymes like endoplasmic reticulum MAN1B1, Golgi MAN1A2 and MAN1C1. However, this fact does not diminish the relevance of MAN1A1 but rather accentuates the interesting potential of α-mannosidases as therapeutic targets, which can be inhibited by available substances such as kifunensine. Thus, α-mannosidases represent an attractive target, since their inhibition might lead to an aberrant N-glycosylation of those CAMs responsible for tumour cell aggregation thereby affecting their function and/or cellular localisation. For OvCa patients this disrupting effect on ascites tumour cell clusters might result in enhanced chemosensitivity and longer RFS and OAS.

One of these CAMs might be ALCAM, which is known to promote tumour progression by affecting diverse cellular functions including tumour cell aggregation. Here, disruption of ALCAM-ALCAM interactions by blocking with a soluble recombinant ALCAM-Fc has been shown to reduce aggregation in different cell lines.^29^ In our study, inhibition of α-mannosidases with kifunensine led to a dose-dependent mass shift of ALCAM in OvCa cells and an accumulation of this protein in the cytoplasm thereby potentially affecting its adhesive properties. The subcellular localisation of ALCAM has been previously described to impact the survival of OvCa patients.^30^ Several studies have shown that ALCAM is increasingly shed by proteases in aggressive OvCa, resulting in various sALCAM forms in the extracellular space.^31^ With our western blot approach, we probably not only detected and quantified full-length ALCAM, but also the larger sALCAM form (ca. 95 kDa). Smaller sALCAM forms of (30 kDa, 60 kDa) were not included in our analysis. The influence of N-glycosylation on ALCAM shedding and on the function of sALCAM warrants further investigation.

In order to analyse if MAN1A1 expression also has an effect on the prognostic role of adhesion proteins in OvCa, we chose ICAM-1 and ALCAM, which were shown to correlate with RFS and OAS in breast cancer in prior studies.^32,33^ In ovarian tumours, our first analysis did not reveal any prognostic significance for these CAMs and stratification for MAN1A1 levels did not show any effect in the case of ICAM-1. In contrast, ALCAM turned out as a significant adverse prognostic indicator in the presence of high (>median) MAN1A1 expression, whereas it was even associated with longer RFS in cases with low-MAN1A1 levels. This confirms our prior results indicating that the biological role of ALCAM is dependent on its N-glycosylation.

Strikingly, MAN1A1 seems to play opposite roles in metastasis of breast and ovarian cancer: High MAN1A1 expression is associated with less metastasis to lymph nodes as well as brain, lung and bone in breast cancer,^9^ whereas it correlates with more distant metastasis in OvCa. This discrepancy might be explained by the different modes of metastasis in both tumours, because peritoneal and hematogenic spread involve different mechanisms and adhesion proteins.

In conclusion, the results of our present study show an oncogenic role of the Golgi mannosidase MAN1A1 in ovarian cancer, where MAN1A1 expression levels significantly affects RFS and OAS. Mannosidase-mediated N-glycosylation has a strong impact on tumour cell aggregation and affects the prognostic impact of the adhesion molecule ALCAM. Thus, the understanding of mannosidase-mediated glycan alteration may provide new options for therapeutic intervention.

## Supplementary information

Supplementary Information

## Data Availability

All data generated or analysed during this study are included in this article and its supplementary information files (available at the British Journal of Cancer’s website).
