# Supplementary material for: Prognostic relevance of the Golgi mannosidase MAN1A1 in ovarian cancer: impact of N-glycosylation on tumour cell aggregation
Source: Br J Cancer. 2019 Oct 29;121(11):944–53. doi: 10.1038/s41416-019-0607-2 (PMC6889143; doi:10.1038/s41416-019-0607-2)
Supplement: Supplementary file 1 — Supplementary Information [file 41416_2019_607_MOESM1_ESM.pdf]

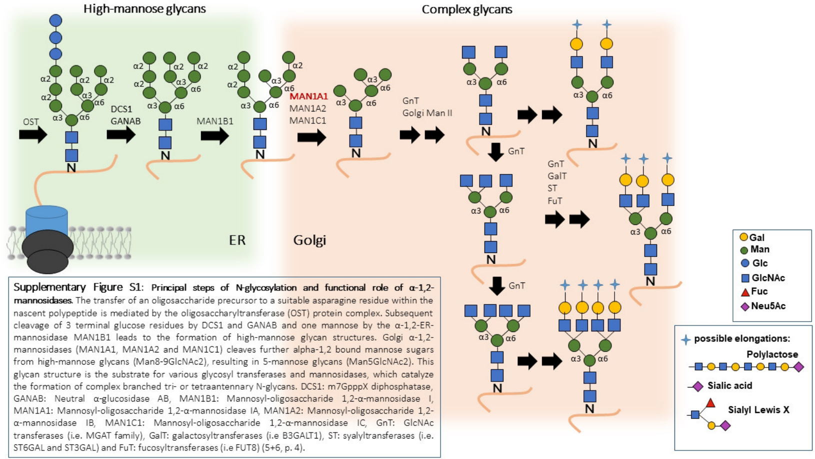

Supplementary Figure S1: Principal steps of N-glycosylation and functional role of α-1,2-mannosidases. The transfer of an oligosaccharide precursor to a suitable asparagine residue within the nascent polypeptide is mediated by the oligosaccharyltransferase (OST) protein complex. Subsequent cleavage of 3 terminal glucose residues by DCS1 and GANAB and one mannose by the α-1,2-ER-mannosidase MAN1B1 leads to the formation of high-mannose glycan structures. Golgi α-1,2-mannosidases (MAN1A1, MAN1A2 and MAN1C1) cleaves further alpha-1,2 bound mannose sugars from high-mannose glycans (Man8-9GlcNAc2), resulting in 5-mannose glycans (Man5GlcNAc2). This glycan structure is the substrate for various glycosyl transferases and mannosidases, which catalyze the formation of complex branched tri- or tetraantennary N-glycans. DCS1: m7GpppX diphosphatase, GANAB: Neutral α-glucosidase AB, MAN1B1: Mannosyl-oligosaccharide 1,2-α-mannosidase I, MAN1A1: Mannosyl-oligosaccharide 1,2-α-mannosidase IA, MAN1A2: Mannosyl-oligosaccharide 1,2-α-mannosidase IB, MAN1C1: Mannosyl-oligosaccharide 1,2-α-mannosidase IC, GnT: GlcNAc transferases (i.e. MGAT family), GalT: galactosyltransferases (i.e B3GALT1), ST: syalyltransferases (i.e. ST6GAL and ST3GAL) and FuT: fucosyltransferases (i.e FUT8) (5+6, p. 4).

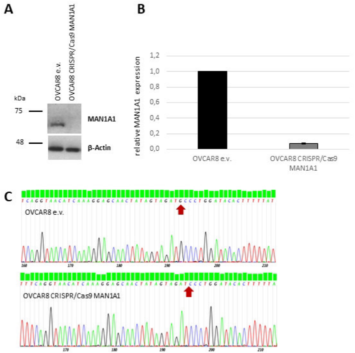

Supplementary Figure S2: Western Blot (A) and PCR (B) analysis showing MAN1A1 knock out in the OVCAR8 cell line (CRISPR/Cas9-MAN1A1) compared to empty vector (e.v.,CRSPR/Cas9). C: DNA-sequencing data showing a single base deletion (arrow) in OVCAR8-MAN1A1 k.o. cells.

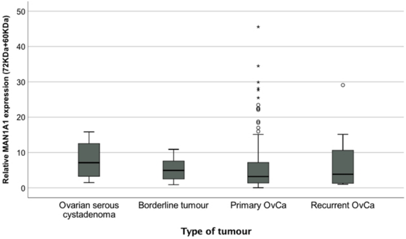

Supplementary Figure S3. Box plot showing MAN1A1 expression (72 kDa + 60 kDa) in different tumour types.

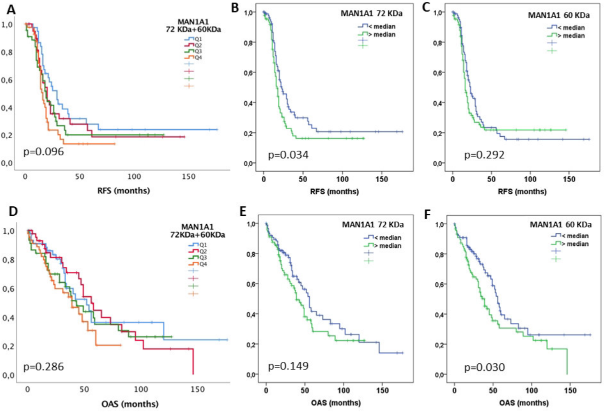

Supplementary Figure S4: Kaplan-Meier analysis showing associations of the different MAN1A1 isoforms (60 KDa and 72 KDa) with recurrence-free (A, B and C) and overall survival (D, E and F) in ovarian carcinomas.

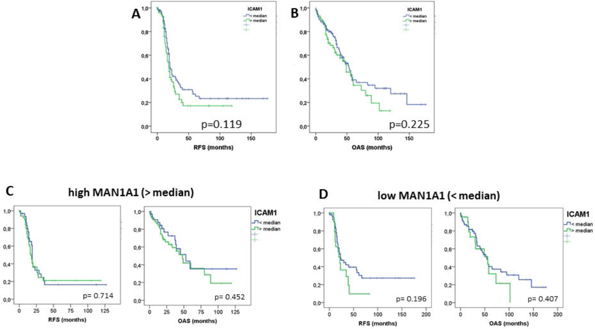

Supplementary Figure S5. Kaplan-Meier analysis showing associations of ICAM-1 expression with recurrence-free (A) and overall survival (B) in ovarian carcinomas in the whole cohort and stratified for low (< median, D) or high (> median, C) MAN1A1 mRNA expression.

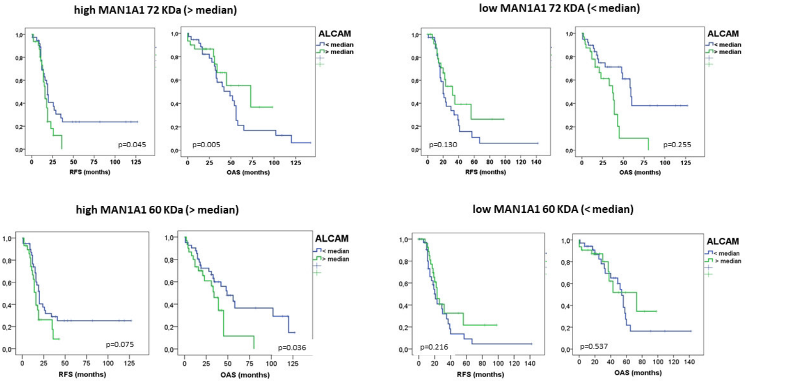

Supplementary Figure S6: Kaplan-Meier analysis showing associations of ALCAM expression with recurrence-free and overall survival in ovarian carcinomas, stratified for low (< median) or high (> median) MAN1A1 mRNA expression of both 72 KDa and 60 KDa isoforms.

Supplementary Table S1: Correlations of MAN1A1 protein expression with clinical and histological tumor parameters[1]

		MAN1A1 (72kDa)			MAN1A1 (60kDa)			MAN1A1 (72kDa+60kDa)		
		< median	> median	p=	< median	> median	p=	< median	> median	p=
Clinical stage	FIGO I-II	12	2		8	6		12	2	
	FIGO III	64	61		67	58		63	62	
	FIGO IV	12	25	**0.003**	13	24	0.122	13	24	**0.005**
Grading	G1-2	26	29		26	29		28	27	
	G3	60	57	0.624	59	58	0.700	58	59	0.870
Lymph node involvement	N0	26	18		22	22		29	15	
	N1	48	53	0.200	51	50	0.956	42	59	**0.007**
Distant metastasis	M0	75	63		74	64		74	64	
	M1	12	25	**0.018**	13	24	**0.046**	13	24	**0.046**
Residual tumor after surgery	no macroscopically visible tumor	70	52		67	55		65	57	
	< 1 cm	13	20		13	20		16	17	
	> 1 cm	5	14	**0.015**	8	11	0.211	7	12	0.397

1, missing values to n=176: no information

Supplementary Material and Methods

Antibodies

Antibody	Company, Catalog No.	Dilution	Incubation
GAPDH	Santa Cruz Biotechnology, Heidelberg, Germany, sc-25778	1:5000 in 1.5 % milk powder in TBS-T	1 hour at room temperature
anti-α-1,2-mannosidase-IA	Abcam, Cambridge, UK, ab140613	1:1000 in 1.5 % milk powder in TBS-T	Over night at 4°C
ALCAM/CD166	Novocastra, Newcastle upon Tyne, UK, NCL-CD166	1:400 in 1.5 % milk powder in TBS-T	Over night at 4°C
ICAM1	Santa Cruz Biotechnology, sc-8439	1:2000 in 1.5 % milk powder in TBS-T	Over night at 4°C
Integrin β4	Santa Cruz Biotechnology, sc-55514	1:400 in 1.5 % milk powder in TBS-T	Over night at 4°C
β- Actin	Santa Cruz Biotechnology, sc-47778	1:2000 in 1.5 % milk powder in TBS-T	1 hour at room temperature
α-Tubulin	Cell Signalling Technology, Cambridge, UK, #2125	1:10000 in 5 % BSA in TBS-T	1 hour at room temperature
Lamin A/C	Cell Signalling Technology, #2032,	1:1000 in 1.5 % milk powder in TBS-T	Over night at 4°C
ConA	Sigma-Aldrich Chemie GmbH, Hamburg, Germany, #C2272, working concentration: 0.025 µg/ml	prepared in Hanks solution (Biochrom GmbH, Berlin, Germany) with 1 mM $MgCl_2$ and 1 mM $CaCl_2$	30 minutes at room temperature
PHA-E	Vector Laboratories, #B-1125, working concentration: 0.5 µg/ml		1 hour at room temperature

TBS-T Buffer: 20 mM Tris-HCl (pH 7.6), 0.137 M NaCl, 10 % Tween-20

Primer sequences for CRISPR/Cas9 MAN1A1 Knock out

sgRNA top: *MAN1A1*_e4_g1_FP: 5´-CACCAGCAACTATAGTAGATGCCC- 3´

sgRNA bottom: *MAN1A1*_e4_g1_RP: 5´-AAACGGGCATCTACTATAGTTGCT- 3´

Quantitative reverse transcriptase polymerase chain reaction

RNA was isolated with RNeasy Mini Kit (Qiagen, Hilden, Germany) and was reverse transcribed using Maxima First Strand cDNA Synthesis Kit (ThermoFisher Scientific; Pinneberg, Germany). Quantitative reverse transcriptase polymerase chain reaction (qRT–PCR) was performed with SYBR Premix Ex Taq (Takara, Kusatsu, Japan) using the Light Cycler (Roche, Mannheim, Germany). We used the following primer for *MAN1A1*: forward primer, 5´-GAAGGCATGGCCCAACACT-3´; reverse primer, 5´-GTAGCGATGGCTTCAACACC-3´. The data were analysed based on the ΔΔCt method as described (Oliveira-Ferrer et al, 2014).

Genomic DNA isolation and Sanger sequencing

Isolation of genomic DNA was performed using QIAamp DNA Mini Kit (Qiagen) according to manufacturer's instruction. For amplification of the genomic region targeted by the CRISPR/Cas9, PCR from genomic DNA of control cells (e.v) and MAN1A1 knock out cells and following primers (exon 4) was performed: forward primer, 5´-TATCAGTCGATAGCCCGTGA-3´; reverse primer, 5´-GGGCCCACTACAGAATTTATCCA-3´. Sanger sequencing analysis was done by TGCA company.
